# Transitioning youth to adult age also through health services

**DOI:** 10.1017/S2045796019000842

**Published:** 2020-01-09

**Authors:** Maurizio Bonati

**Affiliations:** Laboratory for Mother and Child Health, Department of Public Health, Istituto di Ricerche Farmacologiche Mario Negri IRCCS, Via Negri M. 2, 20156 Milan, Italy

**Keywords:** Adolescence, chronic care, health service research, interdisciplinary relations

The developmental transition epoch of late adolescence to early adulthood is a time of evolution (Chan *et al*., 2019). Transition is not synonymous with transfer. Transition is an active action, collegiate and participated. Transfer is a passive action – a suffered one. Transition is the process of preparation for the transfer between paediatric and adult age, and it can also happen for the passage between health services. Transition includes the initial planning, the transfer itself and the support provided throughout, including the support provided in adult care (Schor, 2015). Transition should be a purposeful and planned process provided as a core component of developmentally appropriate health care for all young people with chronic diseases during adolescence and young adulthood (Tuchman *et al*., 2008; Hepburn *et al*., 2015). Unfortunately, only about half of adolescents and young adults with chronic diseases receive any preparation for this transfer of healthcare (Reiss *et al*., 2005), despite the fact that significant determinants influencing gaps in care for adolescents with complex chronic conditions transitioning to adulthood have been identified (Goossens *et al*., 2016). Transition to adult services is a risky period that can lead to reduced engagement with health care, and preventing adolescents from becoming lost in the transfer is one of the challenges (Willis and McDonagh, 2017; Nguyen *et al*., 2018). These challenges are compounded by both multiple, concurrent developmental transitions that may be underway (i.e., shift to independent living, post-secondary education or the workforce and personal and peer relationships/social networks) and a lack of continuity of care into adult services (Suris and Akre, 2015). To complicate things further is the WHO's definition of young people (10–24 years old), increasingly used to reflect the protracted nature of transition as well as the biopsychosocial stages of development (Schor, 2015; Ki-moon, 2016). This, in turn, raises the issue of whether the challenges of transition are more closely related to adolescent and young adult development rather than to the professional handover of care (McManus *et al*., 2015; Leyenaar *et al*., 2017).

Despite the increased interest over the past decade, this issue is addressed by only a few countries (USA, UK and Canada) and is contemplated for specific chronic conditions (diabetes and spina bifida) and with interventions of low documented evidence (Mora *et al*., 2019). Although there has been abundant literature outlining agreed principles of good transitional care, also incorporated into guidelines, the effectiveness has so far been scarce (Nagra *et al*., 2015).

Studies evaluating chronic conditions identified various factors involved in a successful transition, including the importance of continuity and relationships with familiar health professionals and better information and involvement in care management. Studies exploring this issue show that there are often differences between user, parent and clinician perspectives (Reiss *et al*., 2005). The transition to adult services often results in poor patient and parent satisfaction and loss to follow-up for young adults with chronic diseases. (Leyenaar *et al*., 2017; Chu *et al*., 2015; Suris *et al*., 2017). The experiences of young people, parents and clinicians suggest joint-working as a frequent, shared need, given the reported lack of two-way communication as a major impediment to a successful transition process. Moreover, flexibility concerning transition-age thresholds is seen as a key component of good transition by both patients and parents. Studies evaluating parents' perspectives show that parents would like to be more involved in their child care as the child transitions to an adult service, and feel left out or feel they have no one with whom to discuss their worries about their children (Leyenaar *et al*., 2017). This specific need could be related to the different cultural philosophies between child and adult systems, with the first being more family-oriented, inclusive and holistic than adult services, and the second being focused more exclusively on the individual (Davis *et al*., 2014).

Unmet needs for youths moving from paediatric to adult services have been identified across disciplines (i.e., neurology, psychiatry, gastroenterology, rheumatology, pneumology) underling that differences in managing the care and in-service organisation, both between and within countries, accentuate the problems (Benchimol *et al*., 2011; Srivastava *et al*., 2012; Stagi *et al*., 2015; Andrade *et al*., 2017; Mori, 2018). Moreover, chronic disorders belong to a set of comorbidities that can also affect the transition between health services (Van Cleave *et al*., 2013). Thus, although certain programmes have shown positive results within one or a few centres, or in interventional research, their feasibility in daily practice and their adoption at national levels have yet to be defined and reached. Efforts are therefore needed at different levels and should involve all pertinent figures, by relevance and responsibility, to ensure the seamless continuity of care. Supporting youth transitioning into adulthood through integrated and coordinated care is a pressing issue for many countries globally as it impacts research, clinical practice and policy. Thus, determining strategies and appropriate practices to optimise services for transitioning youth and young adults with chronic needs are a priority for healthcare systems.

The following essays offer constructive reflections on the transfer and/or transition of young people with mental disorders, in particular on the currently available evidence for the two most investigated conditions (Attention deficit hyperactivity disorder ADHD and Autism spectrum disorder ASD) in the mental health field. These two disorders differ from chronic conditions for their complexity, comorbidities, multimodal therapy and need for a broad range of services and/or support (Bennett *et al*., 2018; Wilens *et al*., 2018). They are disorders in which the grade of impairment may also affect the event of becoming autonomous to some extent, although this event is the goal of transition of all youths, both healthy and ill, and the expectation of all parents. These are reflections that cannot be exempted from consideration of the social and economic circumstances, the local health-care system and the cultural level of the community where the transition must happen (i.e., where it will be planned, sustained and accompanied) (Eilenberg *et al*., 2019). Thus, transition can also be a determinant of health inequalities within and between countries where patients and parents are too often left alone and can become lost in transition. Consequently, permanent monitoring and implementation actions at local and national levels should be set up involving all the care providers. In addition, future research should focus on providing clear evidence on the effectiveness of transition interventions in all countries.

In the two following essays a few considerations and references are repeated. It would have been possible to edit the text in order to avoid duplication. Instead, the choice was to maintain the repetitions to emphasise the relationship of the two disorders, often as comorbidities, but also to underline possible common answers to these common needs. This, of course, is a reflection to be verified also in many other circumstances.
